# Human perception of AI-generated post-treatment orthodontic facial images: factors associated with misclassification

**DOI:** 10.1007/s00784-026-07020-5

**Published:** 2026-07-15

**Authors:** Gil Guilherme Gasparello, Otso Tirkkonen, Lucas Arrais Campos, Orlando Motohiro Tanaka

**Affiliations:** 1 Graduate Dentistry Program, Orthodontics, Pontifícia Universidade Católica do Paraná – PUCPR School of Medicine and Life Sciences, Rua Imaculada Conceição, 1155, Curitiba, PR CEP 80215-901 Brazil; 2 Research Unit of Population Health, University of Oulu, Oulu, Finland; 3 The Wellbeing Services County of North Ostrobothnia, Oulu, Finland; 4 Faculty of Health Sciences, Institute of Dentistry, University of Eastern Finland, Kuopio, Finland; 5 Faculty of Medicine and Health Technology, Tampere University, Tampere, Finland

**Keywords:** Artificial intelligence, Image perception, Digital trust, Aesthetic judgment, Synthetic media, Orthodontics

## Abstract

**Objectives:**

This study evaluated participants’ ability to differentiate AI-enhanced orthodontic outcome images from real post-treatment images, and examined the demographic and behavioral factors associated with detection accuracy and with perceived attractiveness.

**Materials and methods:**

In a cross-sectional online survey (*N* = 252), participants viewed three sets of smile images—pre-treatment, real post-treatment, and AI-enhanced outcomes generated via ChatGPT with DALL·E 3—and rated each image’s authenticity and attractiveness using a Visual Analogue Scale (VAS). A custom seven-item questionnaire assessed AI use and trust across daily and professional contexts. Generalized estimating equations were used to account for repeated image evaluations within participants, with logistic GEE models for misclassification outcomes and Gaussian GEE models for attractiveness ratings.

**Results:**

A total of 63.2% of participants misclassified AI-enhanced images as real, whereas 18.5% misclassified real images as AI-generated. AI-enhanced images received significantly higher attractiveness ratings (mean VAS = 69.2; 95% CI: 67.5–70.9) than actual post-treatment results (mean VAS = 53.9; 95% CI: 51.9–56.0; *p* < 0.001). Greater trust in AI-generated content was associated with higher odds of misclassifying AI-enhanced images as real (OR = 1.38; 95% CI: 1.13–1.69), and older age showed a small association in the same direction (OR = 1.02; 95% CI: 1.00–1.05).

**Conclusions:**

In this sample, AI-generated orthodontic images were frequently misclassified as real and were perceived as more attractive than real post-treatment photographs. As generative AI tools become more accessible, understanding how demographic and behavioral factors affect human trust and perception is critical for developing responsible AI policies and digital literacy interventions.

**Clinical relevance:**

Clinicians should be aware that AI-generated smile simulations may create unrealistic patient expectations. This study provides empirical evidence supporting the need for transparent patient communication regarding the limitations of AI-generated content in orthodontic practice.

**Supplementary Information:**

The online version contains supplementary material available at 10.1007/s00784-026-07020-5.

## Introduction

Anticipating the transformative potential of machine intelligence, Alan Turing famously proposed a test to evaluate whether a machine could convincingly imitate human communication through text, thereby deceiving a human evaluator into believing they were interacting with another person [1]. Decades later, rapid advances in computing have validated the foresight of Turing’s proposition, as modern generative artificial intelligence (AI) systems increasingly blur the distinction between human and machine capabilities. The proliferation and continual refinement of generative AI, particularly large language models such as ChatGPT and Gemini, have prompted growing concerns about their potential to facilitate academic dishonesty and deceptive self-presentation [2–4], and accelerate the spread of disinformation [5, 6].

Those recent advancements in AI have significantly enhanced the realism of synthetic facial images, raising questions about human perception and trust in AI-generated content, particularly in aesthetic domains such as dental and facial attractiveness [7]. Studies show that people often struggle to distinguish between real and AI-generated images, with misclassification rates approaching chance levels [8, 9]. In some cases, synthetic faces are perceived as more authentic than actual human faces, a phenomenon referred to as AI hyperrealism [10, 11]. This challenges the assumption that photorealistic visuals inherently convey trustworthiness or authenticity. Models of facial attractiveness suggest that computational factors, such as symmetry, proportion, and averageness, strongly influence human judgments of beauty [12]. However, AI-generated beauty standards frequently reflect sociocultural and racial biases, often favoring White or Eurocentric features, which may distort perception of natural diversity [13, 14]. Furthermore, research has shown that faces rated as more attractive are less likely to be classified as AI-generated, suggesting a perceptual bias where beauty is implicitly associated with authenticity [15].

Within clinical contexts such as aesthetic dentistry and orthodontics, AI technologies are increasingly used to simulate and enhance smile outcomes [16, 17]. The shift from occlusion-driven to face-driven orthodontics has accelerated the adoption of digital tools for treatment planning, including AI-powered facial analysis and smile simulation [18, 19]. Tools such as Invisalign’s SmileView and other AI-based digital smile design platforms can generate smile simulations, and studies show that these simulations can improve patients’ aesthetic perception of their smile and, in some cases, increase motivation to pursue orthodontic treatment [20, 21]. However, the predictability of these tools remains an active area of investigation. A prospective clinical trial demonstrated that while some quantitative smile parameters showed no statistically significant difference between simulated and actual post-treatment outcomes, qualitative variables such as lower incisor display showed minimal agreement, indicating that AI smile simulations are not fully reliable predictors of clinical outcomes [22].

This discrepancy raises significant ethical concerns. If AI-generated images are consistently rated as more attractive and realistic than actual clinical results, this may lead to misaligned patient expectations and a potential erosion of trust in real clinical work [23, 24]. The accessibility and simplicity of AI-driven image generation tools have significantly lowered the barrier for creating hyper-realistic facial images [25]. With minimal technical expertise, users can produce visually optimized outcomes by inputting simple prompts, enabling rapid generation of idealized facial representations that align with culturally reinforced beauty standards [26]. As a result, the widespread availability of such tools may contribute to the normalization of digitally perfected appearances that do not necessarily reflect reality or what is attainable even with treatments [8].

Factors such as age, educational level, and familiarity with AI appear to modulate detection ability. Research suggests that older adults may be more susceptible to misclassifying AI-generated images, potentially due to a decline in sensory acuity or greater trust in digital information [27, 28]. Conversely, experience and digital literacy, such as frequent use of AI in academic or professional contexts, may improve detection accuracy [2]. Understanding these influences is a small but crucial step toward an environment where ethical concerns related to AI-generated content can be effectively addressed.

Despite these implications, few empirical studies have examined whether individuals can effectively differentiate between real and AI-enhanced dental images and explored how usage patterns and trust in AI influence perception and aesthetic evaluation. Therefore, the present study aimed to examine participants’ ability to differentiate between AI-enhanced and real orthodontic outcomes, and to identify the demographic and behavioral factors associated with detection accuracy and with the perceived attractiveness of both image types.

## Materials and methods

### Study design

This was a cross-sectional observational study with a non-probabilistic sample. This study was approved by the Research Ethics Committee of the Pontifícia Universidade Católica do Paraná, Brazil, under approval number 6.861.105, CAAE: 77741824.5.0000.0020. Participation was entirely voluntary, responses were collected anonymously, and no personal or identifiable information was recorded or stored at any point. Participants were informed about the study’s objectives and provided their consent before starting the questionnaire, following established ethical guidelines for anonymous online research. Additionally, written consent was obtained from all individuals depicted in the clinical facial photographs, allowing their use as visual stimuli for research purposes. This study adhered to the ethical principles outlined in the Declaration of Helsinki and its subsequent amendments.

### Image preparation

Three full-face clinical photographs, each from a different patient (one photograph per patient), were selected from the patient archive of a private dental clinic. Written consent was obtained from all individuals depicted in the photographs, allowing their use as visual stimuli for research purposes. Because full-face photographs may allow recognition of the individuals depicted, these images are not made available in a public data repository. A panel of five orthodontic specialists, each with over five years of clinical experience, conducted the selection. All photographs were originally taken using a Canon EOS Rebel T7 DSLR camera with a 100 mm macro lens and a ring flash to ensure consistent lighting and high-resolution image quality. The images were captured following a standardized photographic protocol: patients stood in front of a neutral white background, in a natural head position, with the camera positioned 1.5 m away at the height of the patient. Patients were instructed to maintain a natural smile with slightly parted lips, allowing full visibility of the anterior teeth without forced stretching. De-identified versions of the visual stimuli are provided as Supplementary Material 1.

For each case, three images were prepared: (1) the original pre-treatment photograph; (2) the post-treatment photograph following the completion of orthodontic treatment with fixed appliances; and (3) an AI-generated outcome image, created using ChatGPT’s image generation functionality with the prompt: “Create a perfect and realistic smile.” The first image generated by ChatGPT was selected.

### Questionnaire development

This step aimed to develop an exploratory questionnaire with indicators (items) of AI use and trust, particularly in the context of evaluating real and AI-generated outputs. The goal was to explore how participants interact with AI tools in their daily lives, including tasks related to education, professional activities, creative production (e.g., text and image generation), and their level of critical engagement and trust in AI outputs.

The questionnaire items were developed based on a review of relevant literature on AI acceptance and digital trust and refined through an expert consensus process. A panel of five professionals with advanced academic training and research experience in digital health, behavioral sciences, and dentistry participated in three structured online meetings. As a result, seven items were selected to represent key domains of AI interaction. Each item was presented on a 5-point Likert scale, ranging from 0 (“Totally disagree”) to 4 (“Totally agree”). The items were: (1) I use AI to help with everyday tasks; (2) I use AI to help me work/study; (3) I use AI to write texts; (4) I use AI to create images; (5) I trust the answers of AI in all my tasks; (6) I use AI to generate texts, without checking if the information is correct; and (7) I send texts to other people generated by AI without giving the source.

These items were presented to participants in their native language (Portuguese). The items were translated following a forward–backward translation procedure in accordance with established cultural adaptation guidelines [29]. An independent bilingual translator performed the back-translation, and discrepancies were resolved to ensure semantic and conceptual equivalence between versions. The original questions are provided as Supplementary 2.

**Fig. 1:**
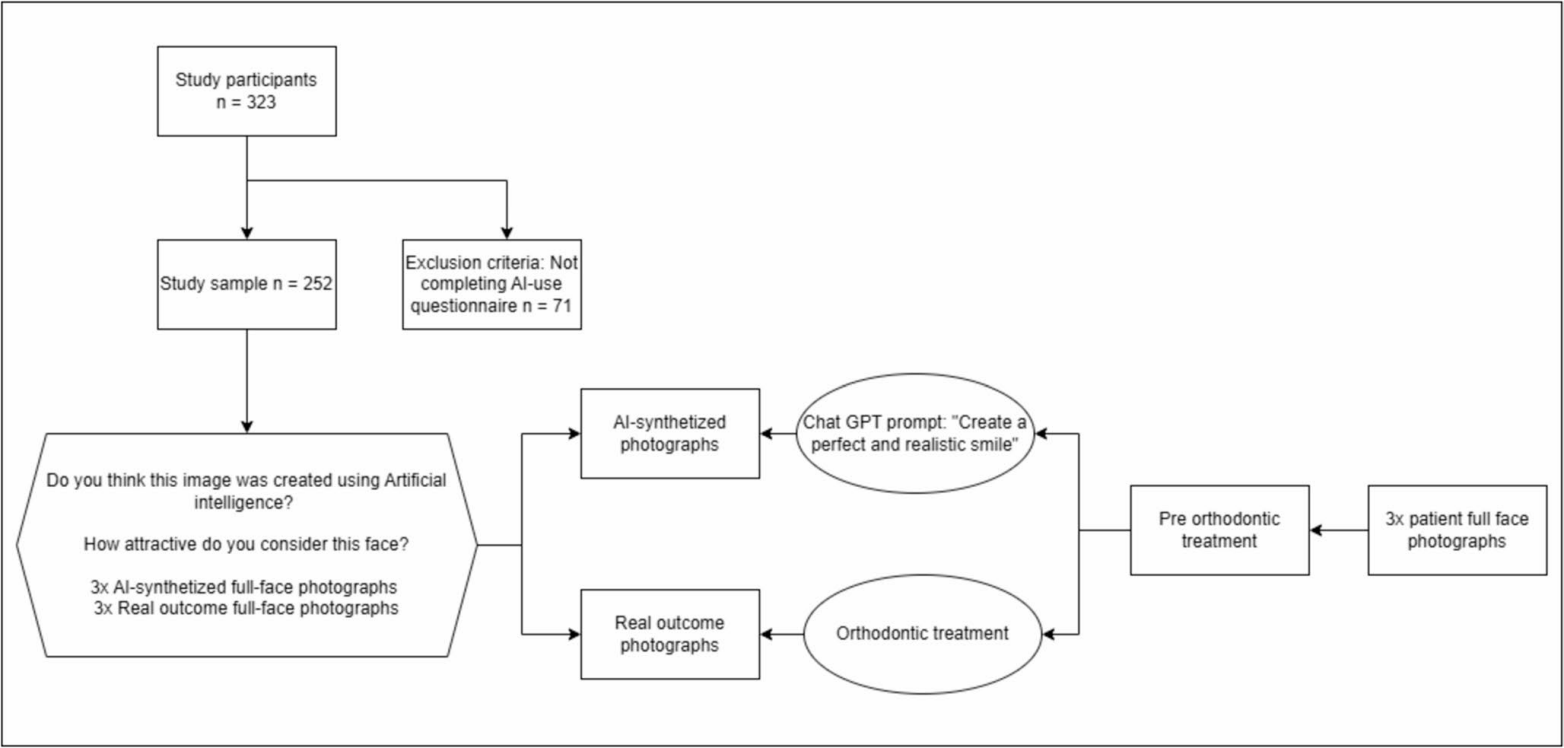
Study design and participant flowchart. A total of 252 participants evaluated three clinical image sets, each comprising pre-treatment, real post-treatment, and AI-enhanced outcome images

In addition, for each image, participants were asked to answer two questions. First, an identification task: “Do you believe this image was created by Artificial Intelligence?” (binary choice: Yes/No). Second, an attractiveness rating: “On a scale from 0 to 100, how attractive do you consider this face?” (Visual Analogue Scale [VAS] ranging from 0 [Not attractive at all] to 100 [Extremely attractive]). Besides evaluating the images, information was collected on participants’ age, sex, and educational level. The study protocol is described more precisely in Fig. 1.

### Sample size calculation

The sample size was calculated assuming an infinite population, a 95% confidence level, and a 7% margin of error, the required minimum sample size was determined using the standard formula for proportions: n = Z² × p × (1 − p) / e², where Z = 1.96 (95%), *p* = 0.5 (conservative), and e = 0.07. The minimum required sample size was 196 participants. The final sample size was 252, with each participant providing evaluations for three outcomes.

### Participants

Participants were sourced from professional networks, dental schools, and social media platforms to ensure a diverse range of backgrounds and familiarity with dental and facial aesthetics. Eligibility criteria included being a resident of Brazil and at least 18 years old. Exclusion criteria were not completing the AI use questionnaire. A total of 323 answers were gathered. After exclusion criteria, the final study sample comprised 252 participants.

### Data collection

Data collection took place via the Qualtrics online survey platform from April 26 to May 8, 2025, focusing on participants living in Brazil. To reduce potential order effects and response bias, the sequence of images within each set was randomized. A flowchart regarding data collection and study procedures is presented in Fig. 1.

### Pilot study

A pilot study involving 40 participants (mean age = 32.6 years, SD = 8.4) was conducted to assess the test–retest reliability of the study instruments. Participants completed the questionnaire on two occasions, separated by a 20-day interval. Test–retest reliability of the VAS scores was evaluated using the intraclass correlation coefficient (ICC), which demonstrated good reliability (ICC = 0.82). For questionnaire items with ordinal response options, agreement between test and retest was assessed using weighted Cohen’s Kappa coefficients, indicating substantial agreement (κw = 0.81, *p* < 0.001). Data from the pilot study were excluded from the final analysis.

### Statistical analysis

All responses were extracted from Qualtrics and analyzed using R software, version 4.4.0 [30]. Descriptive statistics, including frequencies, means, and standard deviations, were calculated. Age, sex, education, and questions related to AI use were considered as factors (independent variables) in this study. Image type was considered as a confounding variable. Student’s paired t-test was utilized to compare VAS scores between real and AI-generated images. Spearman rank correlations of AI use questions were computed using pairwise complete observations. Multicollinearity was assessed using Variance Inflation Factors (VIFs) and adjusted Generalized VIFs (GVIFs) for categorical predictors. All values were below the threshold of 5, indicating no serious multicollinearity [31].

To account for the repeated image evaluations performed by each participant, generalized estimating equations (GEE) were used with participant as the clustering variable and robust standard errors [32]. For attractiveness ratings, Gaussian GEE models were fitted with VAS score as the dependent variable [33]. For misclassification outcomes, logistic GEE models were fitted with misclassification as the dependent variable. Age, sex, education level, AI-use items, and image type were included as independent variables according to each model. For the education variable, the postgraduate (Master’s or Doctorate) category was set as the reference because it represented the highest level of educational attainment, providing a stable baseline against which the performance of participants with lower educational levels could be compared. A p-value < 0.05 was selected as the threshold for statistical significance.

## Results

Participants had a diverse educational background: 33.7% held only a high school diploma, 25.0% had an undergraduate degree, and the remaining participants had higher education qualifications (Table 1). The mean age of participants was 32.9 (SD = 12.3) years, and 65.1% were female. A total of 63.2% of participants misclassified AI-enhanced outcomes as real, while 18.5% misclassified real images as AI-generated. The mean VAS score for AI-enhanced outcomes was 69.2 (95% CI: 67.5–70.9), compared to 53.9 (95% CI: 51.9–56.0) for real outcomes (*p* < 0.001). Descriptive statistics for response items are provided in supplementary 3.

**Table 1:** Descriptive statistics of study variables

Variable	Mean (SD) or % (*n*)
Age	32.9 (12.3)
Sex	
Female	65.1% (164)
Male	34.9% (88)
Education	
High school diploma or equivalent	33.7% (85)
Undergraduate degree	25.0% (63)
Postgraduate degree (Specialization)	22.6% (57)
Postgraduate degree (Master’s or Doctorate)	18.7% (47)
AI Use Items (0–4 Likert scale)	
I use AI to help with everyday tasks (AI_use_1)	2.53 (1.17)
I use AI to help me work/study (AI_use_2)	2.77 (1.21)
I use AI to write texts (AI_use_3)	2.48 (1.35)
I use AI to create images (AI_use_4)	1.83 (1.41)
I trust the answers of AI in all my tasks (AI_use_5)	1.70 (1.22)
I use AI to generate texts without checking accuracy (AI_use_6)	0.96 (1.22)
I send AI-generated texts without citing the source (AI_use_7)	0.71 (1.17)

In the logistic GEE model, only one AI-use questionnaire item was significantly associated with misclassification of AI-enhanced outcomes. (Table 2). Participants who reported greater trust in AI-generated content were more likely to misclassify AI-enhanced outcomes as real (OR = 1.38; 95% CI: 1.13–1.69). Each one-point increase in the Likert scale for trust in AI-generated content was associated with a 38% increase in the odds of misclassifying AI-enhanced outcomes. In addition, older age showed a small and borderline statistically significant association with higher odds of misclassifying AI-enhanced outcomes as real (OR = 1.02; 95% CI: 1.00–1.05). No significant associations were observed for sex, education, or the remaining AI-use variables in relation to misclassification of AI-enhanced outcome images. Regarding real outcomes, none of the AI-use questions were associated with misclassifying real outcomes as AI-generated. However, participants with a high school diploma were significantly less likely to misclassify real outcomes (OR = 0.39; 95% CI: 0.18–0.87).

**Table 2:** GEE logistic regression models evaluating factors associated with misclassification of AI-enhanced and real outcome images

Variable	OR (95% CI) AI-enhanced outcomes	OR (95% CI) Real outcomes
Age	1.02 (1.00–1.05)*	1.01 (0.98–1.03)
Sex		
Female	Ref.	Ref.
Male	1.09 (0.71–1.66)	0.72 (0.42–1.24)
Education		
Postgraduate (Master’s or Doctorate)	Ref.	Ref.
High school diploma	1.21 (0.58–2.54)	0.39 (0.18–0.87)*
Undergraduate degree	1.26 (0.61–2.61)	0.42 (0.18–1.02)
Postgraduate (Specialization)	0.79 (0.40–1.57)	0.71 (0.34–1.49)
AI_use 1	1.05 (0.78–1.39)	0.82 (0.58–1.16)
AI_use 2	0.70 (0.50–1.00)	1.24 (0.82–1.86)
AI_use 3	1.00 (0.81–1.24)	0.99 (0.76–1.28)
AI_use 4	0.97 (0.81–1.16)	1.00 (0.81–1.23)
AI_use 5	1.38 (1.13–1.69)**	0.85 (0.67–1.08)
AI_use 6	0.91 (0.74–1.12)	1.03 (0.80–1.33)
AI_use 7	1.05 (0.83–1.32)	1.07 (0.82–1.41)

All models were adjusted for image type. ***p < 0.001; **p < 0.01; *p < 0.05

Age was the only variable significantly associated with attractiveness ratings for both real and AI-enhanced outcomes (Table 3). Each additional year of age was associated with a 0.50-point increase in VAS scores for real outcomes (95% CI: 0.30–0.70) and a 0.40-point increase in VAS scores for AI-enhanced outcomes (95% CI: 0.21–0.59). No significant associations were observed for sex, education, or AI-use variables with VAS scores for either real or AI-enhanced outcome images. (Table 3).

**Table 3:** GEE linear models evaluating factors associated with VAS scores for AI-enhanced and real outcome images

Variable	Coefficient (95% CI) Real outcome	Coefficient (95% CI) AI-enhanced outcome
Age	0.50 (0.30–0.70)***	0.40 (0.21–0.59)***
Sex		
Female	Ref.	Ref.
Male	0.66 (-4.44–5.76)	-0.99 (-5.59–3.61)
Education		
Postgraduate (Master’s or Doctorate)	Ref.	Ref.
High school diploma	3.00 (-4.73–10.73)	6.81 (-0.37–13.99)
Undergraduate degree	-3.70 (-11.59–4.18)	2.84 (-4.67–10.35)
Postgraduate (Specialization)	-0.15 (-7.69–7.39)	4.46 (-2.45–11.38)
AI_use 1	-0.31 (-3.71–3.09)	-1.36 (-5.44–2.72)
AI_use 2	1.07 (-3.04–5.18)	-1.51 (-5.71–2.70)
AI_use 3	-1.33 (-3.94–1.28)	0.93 (-1.66–3.53)
AI_use 4	-1.02 (-3.18–1.14)	-1.31 (-3.18–0.56)
AI_use 5	0.17 (-2.15–2.49)	1.82 (-0.29–3.93)
AI_use 6	-0.71 (-3.53–2.12)	-0.90 (-3.34–1.53)
AI_use 7	-1.50 (-4.58–1.59)	-0.42 (-2.91–2.08)

All models were adjusted for image type. ***p < 0.001; *p < 0.05

**Fig. 2:**
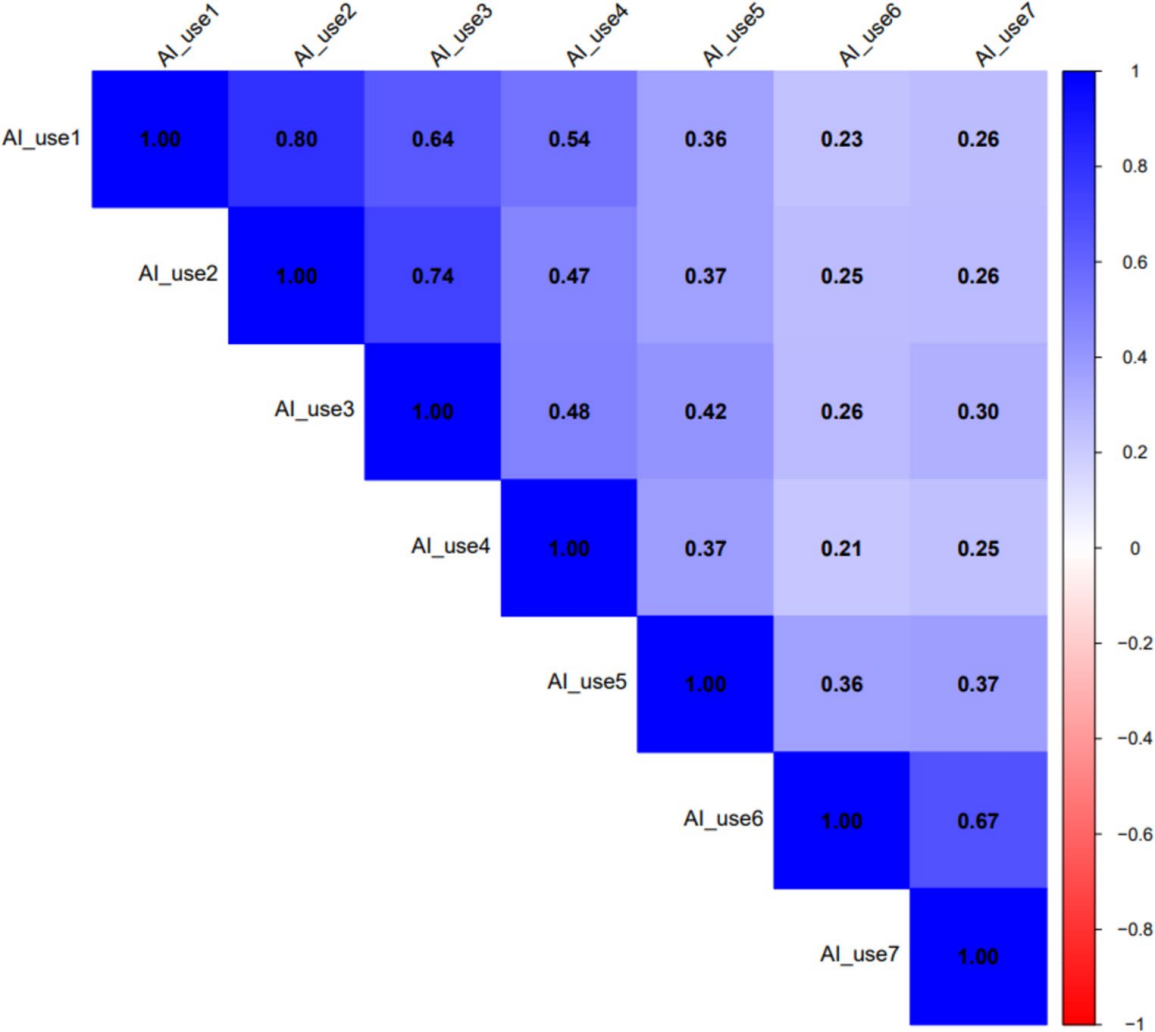
Correlation matrix of the responses to the items of AI-utilization questionnaire

As shown in the correlation matrix (Fig. 2), all items related to AI use were positively correlated with one another. The strongest correlation (*r* = 0.80) was observed between using AI in everyday tasks (Item 1) and using AI for work or study purposes (Item 2). Similarly, generating text with AI (Item 3) was strongly correlated with both Item 1 (*r* = 0.64) and Item 2 (*r* = 0.74). In addition, generating text with AI without checking the source (Item 6) and sending such AI-generated text without giving the source (Item 7) were also strongly correlated (*r* = 0.67).

## Discussion

This study explored participants’ ability to differentiate between AI-enhanced and real orthodontic outcome images and examined the factors associated with perceived smile attractiveness. The study found that older age was associated with both higher aesthetic ratings and a greater likelihood of misclassifying AI-enhanced images as real, Additionally, greater trust in AI-generated answers reduced detection accuracy. These results highlight the role of experience, trust, and age in shaping both the perception and evaluation of AI-generated facial imagery, and also demonstrate that the majority of participants (63.2%) misclassified AI-generated images as real. Furthermore, AI-enhanced images received significantly higher attractiveness ratings (mean VAS = 69.2 compared to real post-treatment images mean = 53.9).

As of today, the primary line of defense against deceptive synthetic media largely relies on the human observer’s perceptual ability to visually or auditorily distinguish AI-generated content from authentic material [34]. However, as the realism of synthetic media increases, this task becomes increasingly difficult. Research has shown that individuals tend to overestimate their ability to detect synthetic content [35, 36]. Given these findings, accurately assessing individuals’ perceptual ability to distinguish between real and AI-generated content is essential for addressing the growing risks associated with synthetic media. Although there is increasing interest in technical solutions, such as machine-based detection systems, watermarking, and content provenance frameworks, these tools remain either insufficiently robust or not yet widely adopted to ensure effective protection at scale [37, 38]. These findings indicate the need for strategies beyond technical detection tools, including educational interventions that strengthen users’ ability to critically evaluate synthetic content as AI-generated images become increasingly realistic.

The present study found that older participants were slightly more likely to misclassify AI-enhanced images as real, although this association was small and close to the threshold of statistical significance. This finding cautiously suggests a possible age-related reduction in detection accuracy and aligns with prior studies showing that older adults may be less accurate in identifying synthetic media across modalities [39, 40]. One possible explanation lies in age-related sensory degradation, such as diminished visual acuity, which may reduce sensitivity to subtle artifacts commonly present in AI-generated content [41]. Moreover, older individuals tend to rely more on multisensory integration to compensate for unisensory deficits [42], which can paradoxically increase vulnerability to deceptive or mismatched synthetic stimuli. Although this finding should be interpreted cautiously, it suggests that older adults may benefit from clearer guidance and more accessible tools to help them evaluate increasingly realistic AI-generated media.

AI-generated faces are often perceived as more visually appealing than real human faces [8, 10], our results indicate that orthodontic AI-enhanced images may elicit similar effects. One study demonstrated that synthetically generated faces were not only indistinguishable from real ones but were also judged as more aesthetic [8]. This finding aligns with prior evidence showing that facial symmetry, smooth texture, and averageness are powerful predictors of attractiveness and positive social evaluation [12, 43]. Notably, in our study, even a single, non-optimized prompt requesting a “perfect and realistic smile” was sufficient to generate AI-enhanced outcomes that were perceived as more appealing than real post-treatment results. This underscores the power of large generative models to exploit perceptual cues associated with beauty, even in the absence of advanced prompt engineering or iterative refinement. In the context of orthodontics, this has direct clinical implications: patients who are exposed to AI-generated smile simulations may develop expectations that exceed what is clinically achievable, potentially leading to dissatisfaction with real treatment outcomes [23, 24].

The finding that greater trust in AI-generated answers was associated with higher odds of misclassifying AI-enhanced outcomes as real is particularly relevant. This suggests that individuals who place more trust in AI outputs may evaluate synthetic or digitally enhanced content with less skepticism, making them more susceptible to accepting AI-enhanced images as authentic. Although trust in AI was not independently associated with attractiveness ratings in the adjusted models, this result has important implications for orthodontic communication. In clinical settings, patients who are highly trusting of AI-generated content may be more likely to accept digitally enhanced simulations as realistic representations of treatment outcomes. Similarly, orthodontists and other clinicians may increasingly use AI tools to search for clinical information, treatment-related explanations, or patient communication materials, although such outputs may not always be accurate, complete, or evidence-based. Orthodontists should therefore be prepared not only to explain the distinction between AI-enhanced images, digital simulations, and clinically achievable results, but also to critically appraise AI-generated information before applying it in clinical communication or decision-making. This interpretation is consistent with broader discussions on digital literacy, which emphasize that critical engagement with technology, rather than passive exposure to it, is essential for developing media discernment skills [2, 28].

Regarding the questionnaire used in this study, it was developed as an exploratory instrument to capture key dimensions of AI use and trust, rather than as a validated psychometric scale. While test-retest reliability was assessed and found to be satisfactory, future research should consider developing and validating more comprehensive instruments to measure AI trust and literacy, particularly in clinical populations. Additionally, the study was conducted in Brazil, and the findings may not be generalizable to other cultural contexts where attitudes toward AI and digital literacy may differ. Future studies should replicate these findings in diverse populations and explore the role of cultural factors in shaping AI perception and trust.

### Limitations

Several limitations of this study should be acknowledged. First, all AI-generated images were produced using a single model (ChatGPT with DALL·E 3) on a single date (April 19, 2025) using fixed prompts. Generative AI technologies vary widely across platforms, and the outputs of a single model can shift significantly over time due to updates or fine-tuning. Second, only three clinical image cases were used as visual stimuli. Although this allowed a standardized comparison between pre-treatment, real post-treatment, and AI-enhanced outcomes, the findings may partly reflect the specific characteristics of these selected photographs rather than the broader perception of AI-enhanced orthodontic outcomes. Future studies should include a larger and more diverse set of clinical cases, malocclusion types, facial patterns, and AI-generation strategies. Third, the study used a non-probabilistic sample recruited through professional networks and social media, which may introduce selection bias. Fourth, the sample was limited to Brazilian residents, which may limit the generalizability of the findings. Fifth, the study design did not allow for the assessment of causal relationships between the variables studied.

## Conclusions

Most participants (63.2%) could not distinguish AI-generated images from real post-treatment photographs, and AI-enhanced images were rated as more attractive. Older age and greater trust in AI were associated with higher odds of misclassification. Clinicians should be aware that AI-generated smile simulations can raise unrealistic patient expectations, and should address the limitations of such content when communicating with patients.

## Supplementary Information

Supplementary Material 1.

Supplementary Material 2.

Supplementary Material 3.

## Data Availability

The anonymized numerical survey dataset generated and analyzed during the current study is available from the corresponding author upon reasonable request and after assessment of ethical and privacy restrictions. Clinical images are not deposited in a public repository due to ethical and privacy considerations related to image-based clinical material and the scope of the original informed consent.

## Declarations

### Generative AI use

Generative AI tools were used to create the AI-generated image stimuli described in the Methods section and for English language refinement. They were not used to generate scientific interpretations, statistical analyses, or conclusions.
