# Supplementary material for: Human perception of AI-generated post-treatment orthodontic facial images: factors associated with misclassification
Source: Clin Oral Investig. 2026 Jul 15;30(8):345. doi: 10.1007/s00784-026-07020-5 (PMC13379457; doi:10.1007/s00784-026-07020-5)
Supplement: Supplementary file 1 — Supplementary Material 1. [file 784_2026_7020_MOESM1_ESM.docx]

Pre-treatment

Post-treatment

AI-generated outcome


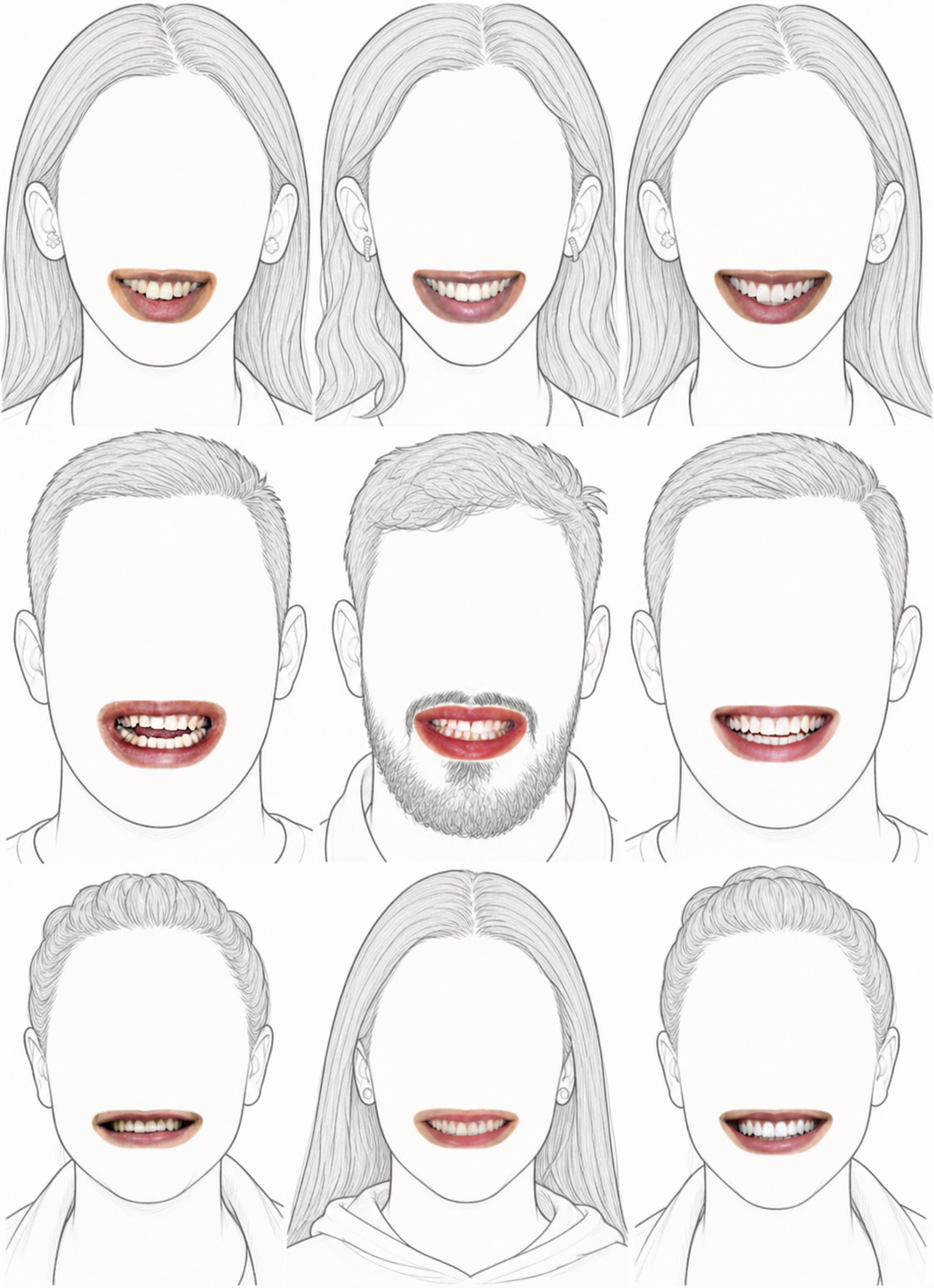


Supplementary Figure S1. De-identified visual stimuli used in the study. Each row corresponds to one case, with pre-treatment, real post-treatment, and AI-generated outcome images shown from left to right. The upper and middle facial thirds were masked using a schematic illustration, while the lower facial third and dental/smile region were preserved to allow visual assessment without exposing identifiable facial features.

Note; To prepare the de-identified supplementary images, the original visual stimuli were edited in Adobe Photoshop. The upper and middle facial thirds, including the eyes, nose, forehead, and other potentially identifiable facial features, were manually masked and replaced with a neutral schematic illustration of the face and hair. The lips, teeth, smile and surrounding perioral region, were preserved from the original images and was not altered, retouched, or enhanced. This procedure was applied consistently to all pre-treatment, post-treatment, and AI-generated outcome images included in the supplementary material. The purpose of this editing was solely to reduce identifiability while preserving the smile-related region relevant to the study.
