# Supplementary material for: Human perception of AI-generated post-treatment orthodontic facial images: factors associated with misclassification
Source: Clin Oral Investig. 2026 Jul 15;30(8):345. doi: 10.1007/s00784-026-07020-5 (PMC13379457; doi:10.1007/s00784-026-07020-5)
Supplement: Supplementary file 2 — Supplementary Material 2. [file 784_2026_7020_MOESM2_ESM.docx]

# Supplementary 2 – Original Questionnaire Items (Portuguese)

The following items were presented to participants in Brazilian Portuguese. An English translation is provided for each item.

## Demographic Information

**Idade (Age):** Qual é a sua idade? / What is your age? [Open-ended numeric response]

**Sexo (Sex):** Qual é o seu sexo? / What is your sex? [Feminino / Masculino / Outro / Prefiro não informar]

**Escolaridade (Education):** Qual é o seu nível de escolaridade? / What is your highest level of education?
[Ensino Médio / Graduação / Pós-graduação (Especialização) / Pós-graduação (Mestrado ou Doutorado)]

## Image Evaluation Questions (repeated for each of the three image sets)

**Identification task:** Você acredita que esta imagem foi criada por Inteligência Artificial? / Do you believe this image was created by Artificial Intelligence? [Sim (Yes) / Não (No)]

**Attractiveness rating:** Em uma escala de 0 a 100, o quão atraente você considera este rosto? / On a scale from 0 to 100, how attractive do you consider this face? [VAS: 0 = Nada atraente (Not attractive at all) – 100 = Extremamente atraente (Extremely attractive)]

## AI Use and Trust Questionnaire Items (5-point Likert scale: 0 = Discordo totalmente / 4 = Concordo totalmente)

**AI_use_1:** Utilizo IA para me ajudar nas tarefas do dia a dia. / I use AI to help with everyday tasks.

**AI_use_2:** Utilizo IA para me ajudar no trabalho/estudo. / I use AI to help me work/study.

**AI_use_3:** Utilizo IA para escrever textos. / I use AI to write texts.

**AI_use_4:** Utilizo IA para criar imagens. / I use AI to create images.

**AI_use_5:** Confio nas respostas da IA em todas as minhas tarefas. / I trust the answers of AI in all my tasks.

**AI_use_6:** Utilizo IA para gerar textos, sem verificar se a informação está correta. / I use AI to generate texts, without checking if the information is correct.

**AI_use_7:** Envio textos para outras pessoas gerados por IA sem dar a fonte. / I send texts to other people generated by AI without giving the source.
