# Supplementary material for: Human perception of AI-generated post-treatment orthodontic facial images: factors associated with misclassification
Source: Clin Oral Investig. 2026 Jul 15;30(8):345. doi: 10.1007/s00784-026-07020-5 (PMC13379457; doi:10.1007/s00784-026-07020-5)
Supplement: Supplementary file 3 — Supplementary Material 3. [file 784_2026_7020_MOESM3_ESM.docx]

# Supplementary 3 – Descriptive Statistics for AI Use and Trust Items

The table below presents the descriptive statistics for the seven AI use and trust items stratified by image classification outcome (correct vs. misclassification). All items were measured on a 5-point Likert scale (0–4).

**Online Resource 2, Table S1** Descriptive statistics for AI use and trust items by classification accuracy

| **Item** | **Description** | **Overall Mean (SD)** | **Correct Mean (SD)** | **Misclassified Mean (SD)** |
| --- | --- | --- | --- | --- |
| AI_use_1 | I use AI to help with everyday tasks | 2.53 (1.17) | 2.61 (1.14) | 2.48 (1.19) |
| AI_use_2 | I use AI to help me work/study | 2.77 (1.21) | 2.98 (1.15) | 2.63 (1.23) |
| AI_use_3 | I use AI to write texts | 2.48 (1.35) | 2.52 (1.33) | 2.45 (1.37) |
| AI_use_4 | I use AI to create images | 1.83 (1.41) | 1.89 (1.40) | 1.79 (1.42) |
| AI_use_5 | I trust the answers of AI in all my tasks | 1.70 (1.22) | 1.45 (1.18) | 1.85 (1.23) |
| AI_use_6 | I use AI to generate texts without checking accuracy | 0.96 (1.22) | 0.91 (1.19) | 0.99 (1.24) |
| AI_use_7 | I send AI-generated texts without citing the source | 0.71 (1.17) | 0.68 (1.14) | 0.73 (1.19) |

Note. SD = Standard Deviation. ‘Correct’ refers to participants who correctly identified the AI-generated image as AI-generated. ‘Misclassified’ refers to participants who incorrectly identified the AI-generated image as real.
